# Comparative Study on the Impact of Different Extraction Technologies on Structural Characteristics, Physicochemical Properties, and Biological Activities of Polysaccharides from Seedless Chestnut Rose (*Rosa sterilis*) Fruit

**DOI:** 10.3390/foods13050772

**Published:** 2024-03-01

**Authors:** Kaiwen Chen, Qiuqiu Zhang, Shengzhen Yang, Shengyan Zhang, Guangjing Chen

**Affiliations:** 1 College of Food Science and Engineering, Guiyang University, 130 Jianlongdong Road, Nanming District, Guiyang 550005, China; chenkaiwen2001@126.com (K.C.); zhangqq1248@163.com (Q.Z.); ysz20000101@163.com (S.Y.); zsy010913@126.com (S.Z.); 2 College of Life Sciences, South China Agricultural University, Guangzhou 510642, China

**Keywords:** seedless chestnut rose, polysaccharides, extraction method, structure, antioxidant, α-glucosidase, inhibitory mechanism

## Abstract

Seedless chestnut rose (*Rosa sterilis* S. D. Shi, RS) is a fresh type of *R. roxburghii* Tratt with copious functional components in its fruit. Polysaccharides are recognized as one of the vital bioactive compounds in RS fruits, but their antioxidant and hypoglycemic properties have not been extensively explored. Hence, in this study, accelerated solvent extraction (RSP-W), citric acid (RSP-C), 5% sodium hydroxide/0.05% sodium borohydride (RSP-A), and 0.9% sodium chloride (RSP-S) solution extraction were individually utilized to obtain RS fruit polysaccharides. The physicochemical properties, structural characteristics, and biological activities were then compared. Results indicated that extraction methods had significant influences on the extraction yield, uronic acid content, monosaccharide composition, molecular weight, particle size, thermal stability, triple-helical structure, and surface morphology of RSPs apart from the major linkage bands and crystalline characteristics. The bioactivity tests showed that the RSP-S, which had the greatest amount of uronic acid and a comparatively lower molecular weight, exhibited more potent antioxidant and α-glucosidase inhibitory property. Furthermore, all RSPs inhibited α-glucosidase through a mixed-type manner and quenched their fluorescence predominantly via a static quenching mechanism, with RSP-S showing the highest binding efficiency. Our findings provide a theoretical basis for utilizing RSPs as functional ingredients in food industries.

## 1. Introduction

In recent years, natural polysaccharides have garnered global interest for their multi-functional biological properties, including antioxidant, immunomodulatory, hypoglycemic, and gastroprotective effects [1,2,3]. This has led to an increased focus on their potential applications in functional foods and medicinal products. In recent years, diabetes mellitus (DM), a metabolic condition marked by increased levels of glucose in the bloodstream, has become a notable concern in terms of public health. [4]. It is one of the leading chronic diseases and causes of mortality in modern times. α-Glucosidase, an enzyme located in the brush border of the small intestinal chorion, plays a crucial role in carbohydrate digestion by breaking down the hydrolysis of terminal α-1,4, α-1,2, α-1,3, and α-1,6 glycosidic linkages of oligosaccharides and disaccharides into simpler, absorbable sugars like glucose and fructose [5]. Elevated glucose levels may cause an unevenness in oxidative pressure and the excessive generation of reactive oxygen species (ROS), consequently resulting in harm to cellular structures and cell death [6]. Inhibiting α-glucosidase can effectively reduce post-meal blood glucose spikes in diabetic individuals, making it a key target in diabetes management [7]. Consequently, the evaluation of antioxidant and α-glucosidase inhibitory activities in natural plant-derived polysaccharides is increasingly common in developing new hypoglycemic drugs or functional foods.

Seedless chestnut rose (*Rosa sterilis* S. D. Shi, RS), also known as Golden Cili, is a perennial deciduous climbing shrub native to Guizhou Province of China, celebrated for its attractive flowers and unique seedless fruits, which are notably high in vitamin C content [8]. This plant was identified over 30 years ago by the Guizhou Botanical Garden as a new variety of *Rosa roxburghii* Tratt (RRT) [9]. RS shares a significant genetic similarity with RRT and predominantly thrives in the hilly mountain regions of Anshun, Guizhou Province. Research has largely concentrated on RRT, revealing its high content of ascorbic acid, polysaccharides, phenolics, and superoxide dismutase. These components suggested its effectiveness in preventing type 2 diabetes, hindering cancer cell growth, and combating atherosclerosis [10,11]. In contrast, studies on RS are relatively scarce, with the focus primarily on its fruit characteristics and nutritional content, including vitamin C, trace elements, amino acids, flavonoids, and triterpenes [12,13,14,15]. Based on the information available to us, there have been few investigations that specifically target the RS polysaccharides (RSPs). A few reports revealed that RSPs exhibit prebiotic activity, demonstrating their potential health benefits [16]. Nevertheless, there exists a significant lack of research concerning their antioxidant and hypoglycemic characteristics, constraining their application in the food industry.

A multitude of techniques have been developed for extracting polysaccharides from plant material. Previous studies have indicated a clear connection between the antioxidant and hypoglycemic properties of polysaccharides and their structure in relation to molecular weight, composition of monosaccharides, and glycosidic linkages, which were notably impacted by the extraction method [17,18]. Consequently, a range of technologies have been employed to isolate polysaccharides, such as hot water, ultrasonic, enzyme-assisted, acidic, chelating agent, and dilute alkaline processes [19,20]. For instance, the extraction of sea buckthorn polysaccharide using an alkali-assisted method, which produced a polysaccharide with the lowest molecular weight and highest concentration of arabinose, demonstrated superior antioxidant ability [21]. According to the report, the blackberry fruit polysaccharide acquired through the use of hot water with a greater molecular weight demonstrated more potent antioxidant and α-glucosidase inhibitory effects [22]. Thus, choosing the right extraction technique is crucial for obtaining bioactive polysaccharides. However, there remains an insufficiency of extensive information regarding the structural attributes, physicochemical properties, and biological functionalities of polysaccharides obtained from RS through various extraction methods.

In addition, every method for extracting polysaccharides has its own set of benefits and limitations, taking into account factors like the intricacy of the substance, the effectiveness of extraction, the cost effectiveness, and the impact on the environment. Ethanol precipitation commonly serves as the initial step in polysaccharide preparation from aqueous extracts. To minimize ethanol use, these extracts typically require concentration before precipitation. This process, however, necessitates prior centrifugation or filtration of the aqueous extracts, adding complexity and time to the extraction procedure, which challenges its scalability for industrial applications. Consequently, widespread adoption of these methods in industrial settings for polysaccharide extraction remains limited. In contrast, accelerated solvent extraction (ASE) emerges as a more efficient solution. The automated method utilizes elevated temperatures and pressures to accomplish effective extraction within a short duration [23]. Elevated pressure maintains the solvents in a liquid state beyond their boiling points, thereby speeding up the extraction process, while the increased temperature accelerates the extraction kinetics and enhances the solubility of polysaccharides [24]. Additionally, ASE streamlines the overall procedure by eliminating the need for steps such as centrifugation, filtration, and concentration, reducing sample handling time and simplifying the process [25]. Therefore, from an industrial perspective, ASE presents a promising alternative, characterized by its high efficiency, time-saving nature, and automated capabilities for extracting polysaccharides from RS. However, it is important to note the current gap in research regarding the use of ASE for polysaccharide extraction from RS, particularly in terms of subsequent characterization studies.

Hence, the current research seeks to elucidate the impacts of four different extraction methods, namely ASE, acidic, alkaline, and NaCl solution extraction, on the yield, structure, physicochemical characteristics, in vitro antioxidant properties, and inhibition of α-glucosidase activities of RS fruit polysaccharides. The findings derived from this research are significant in offering an appropriate approach for extracting RS polysaccharides that can be practically applied in the production of health-promoting foods.

## 2. Materials and Methods

### 2.1. Materials and Chemicals

Fresh seedless chestnut rose (*R. sterilis*) fruits were collected in October 2021 on the farmland of Zhenning, Anshun, located in Guizhou Province, China (26°2′49.42″ N, 105°41′59.45″ E, 1247.6 m above sea level). Standard monosaccharides, 3-Phenylphenol, trifluoroacetic acid (TFA), 1,10-Phenanthroline, 1,1-diphenyl-2-picrylhydrazyl (DPPH), 2,2′-azinobis (3-ethylbenzothiazoline-6-sulphonic acid) (ABTS), and 3-(2-Pyridyl)-5,6-diphenyl-1,2,4-triazine-*p*,*p*′-disulfonic acid monosodium salt hydrate were purchased from TOKYO Chemical Industry Co., Ltd. (Tokyo, Japan). Sodium hydroxide was purchased from Thermo Fisher Co., Waltham, MA, USA. The pullulan polysaccharide calibration kit was purchased from Agilent Co., Santa Clara, CA, USA. α-Glucosidase, *p*-nitrophenyl-α-d-glucopyranoside (PNPG), and serum albumin (BSA) were obtained from Yuanye Bio-technology (Shanghai, China). All additional solvents and chemical reagents were acquired locally and were of either HPLC or analytical quality.

### 2.2. Extraction of RSPs

#### 2.2.1. Pretreatment and Extraction Process of RS Fruits

**Figure 1:**
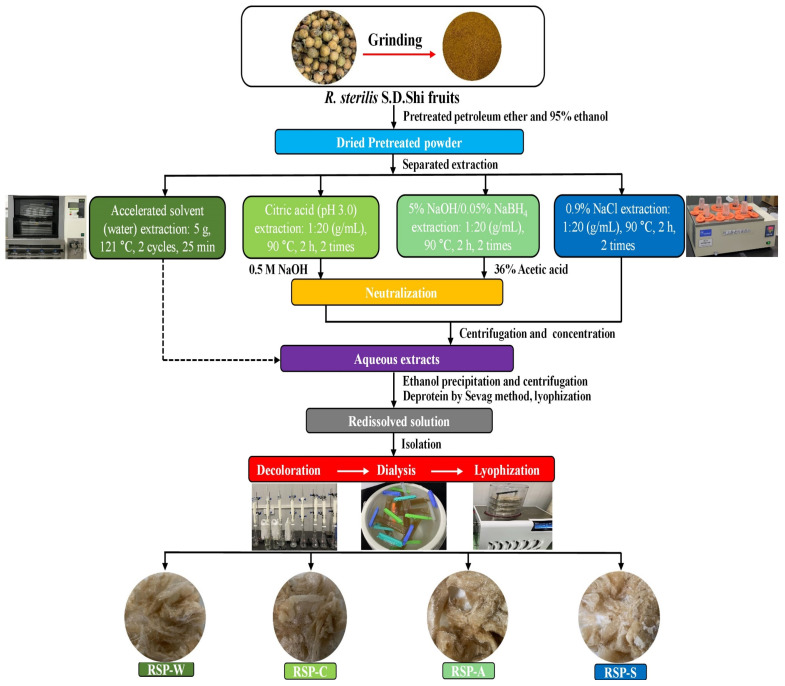
The scheme of polysaccharides extracted from seedless chestnut rose fruit with different extraction technologies. RSP-W, RSPs obtained by accelerated solvent extraction; RSP-C, RSPs obtained by citric acid solution extraction; RSP-A, RSPs obtained by 5% sodium hydroxide/0.05% sodium borohydride solution extraction; RSP-S, RSPs obtained by 0.9% sodium chloride solution extraction.

The RS fruits were collected to the laboratory and stored at 4 °C. Within 2 days, they were washed, oven dried at 55 °C for 48 h, and subsequently ground into fine powders (60 mesh). In order to remove oils and small molecular impurities such as oligosaccharides, monosaccharides, polyphenols, and pigments, the powders underwent three times of treatment using petroleum ether and 95% ethanol (*v*/*v*) at room temperature. After the separation, the resulting residue was dried for 24 h at a temperature of 40 °C and then kept in airtight plastic bags for future use. The comprehensive procedures for extracting and purifying polysaccharides from RS fruit are depicted in Figure 1.

#### 2.2.2. Polysaccharides Extraction

The RS fruit polysaccharides were extracted using four techniques (ASE, citric acid, alkali, and NaCl solutions) based on our previous method with slight modifications [18,26]. The corresponding polysaccharides obtained were named RSP-W, RSP-C, RSP-A, and RSP-S. Our preliminary study ensured that each method was executed with the optimal parameters. The ASE procedure was conducted using a Buchi^TM^ Speed Extractor E-914 system. Initially, five grams of pretreated RS fruit powders were evenly distributed in two separate layers of diatomaceous earth, each layer weighing 2.5 g. The blend was poured into an extraction cell made of stainless steel with a volume of 80 mL, and the extraction of polysaccharides was carried out using distilled water. In order to avoid the entry of suspended particles into the collection vial, the cells were equipped with a stainless-steel frit and a cellulose material. Once distilled water was added, the extraction cells were pressurized, heated, and extracted statically at 100 bar and 121 °C. The cells were purged with nitrogen gas after a static extraction timing of 50 min (consisting of 2 static cycles) and subsequent flushing with distilled water. Afterwards, the water-based extract and cleaning solution, amounting to a combined volume of 42 mL, were collected automatically in glass vials with a capacity of 240 mL. Once the extract cooled to room temperature, it was combined with ethanol to achieve an 80% (*v*/*v*) concentration and left overnight at 4 °C for precipitation. The precipitate was then separated through centrifugation (4 °C, 4500 r/min, 10 min), followed by sequential washes using ethanol, acetone, and anhydrous diethyl ether. The purified solid was dissolved again in distilled water, subjected to deproteinization five times with the Sevag reagent (chloroform to *n*-butanol ratio of 4:1, *v*/*v*), and then dried at −80 °C for 26 h using a vacuum freeze dryer. The vacuum degree was set at 3.5 mbar in accordance with the vapor pressure curve. For further purification, the extracts were dissolved in distilled water again, dynamically decolorized using an HP-20 macroporous resin column (45 × 500 mm), and then dialyzed against distilled water for 48 h using a membrane with a molecular weight cutoff of 8000–14,000 Da. The substance retained in the dialysis bag was ultimately gathered, concentrated, and freeze dried, resulting in the production of the final RSP-W. The yield (%) of RSP-W was calculated as follows:

(1) $$\text{RSP-W}\;\mathrm{yield}\;(\%,\;w/w)=\frac{\mathrm{weight}\;\mathrm{of}\;\mathrm{dried}\;\mathrm{RSP}-W\;(g)}{\mathrm{weight}\;\mathrm{of}\;\mathrm{pretreated}\;\mathrm{RS}\;\mathrm{fruit}\;\mathrm{powder}\;(g)}\times 100$$

For RSP-C, RSP-A, and RSP-S, the pretreated powder (20 g) was mixed with 400 mL citric acid (pH 3.0), 5% NaOH/0.05% NaBH_4_, and 0.9% NaCl, respectively, and the extraction was performed at 90 °C for 2 h (twice). Following centrifugation, the liquid above the sediment of the citric acid and 5% NaOH/0.05% NaBH_4_ water-based solutions was balanced out using either 0.5 M NaOH or 36% acetic acid. Then, the extracted solutions were combined and concentrated, and the next steps were the same as ASE.

### 2.3. Chemical Composition and Monosaccharide Composition Analysis

The quantification of neutral sugar in each RSP was accomplished through the phenol-sulfuric acid method [27], and the results were presented in terms of glucose equivalents. To assess the uronic acid content, the modified *m*-hydroxydiphenyl method was employed, using galacturonic acid as a reference [28]. Additionally, the Bradford assay was applied to determine protein content using bovine serum albumin (BSA) as a standard [29].

The monosaccharide profiles of RSPs extracted via four distinct methods were characterized using high-performance anion-exchange chromatography (HPAEC) following previously established protocols [20]. The RSP samples, enclosed in tubes, underwent hydrolysis with 3 M trifluoroacetic acid (TFA) at a temperature of 120 °C for a duration of 3 h. Afterward, the hydrolyzed RSP samples were evaporated three times using rotary vacuum evaporation and co-distillation with methanol to eliminate TFA. The treated samples, in addition to standard solutions, were dissolved in distilled water and subsequently passed through a 0.22 μm filter for analysis using an ICS-6000 system (Thermo Fisher, Waltham, MA, USA). The system was equipped with a Dionex CarboPac^TM^ PA20 safeguard column (3 mm × 30 mm), a Dionex CarboPac^TM^ PA20 analytical column (3 mm × 150 mm), and an electrochemical detector (ECD). The mobile phase consisted of a 15 mM NaOH solution (A), a 0.25 M NaOH solution (B), and a 15 mM NaOH solution containing 0.1 M NaAc (C), flowing at a rate of 0.3 mL/min. The gradient elution program was set as follows: 0–25 min with 100% A, 25.1–52 min with 100% C, and 52.1–70 min with 100% B. The column temperature was maintained at 30 °C, and the injection volume was 25 μL. Monosaccharide concentrations were quantified based on the calibration curves (peak area–concentration) for each standard monosaccharide.

### 2.4. Molecular Weight Distribution Determination

The weight-average molecular weight (Mw), number-average molecular weight (Mn), and polydispersity index (Mw/Mn) of RSPs were measured by high-performance gel permeation chromatography (HPGPC, Agilent 1260, Agilent Co., Santa Clara, CA, USA) coupled to a refractive index detector (RID, Agilent) at 35 °C. PL aquagel-OH 60 gel column (25 mm × 300 mm, 8 μm, Agilent, Cheadle, UK) and PL aquagel-OH 50 gel column (25 mm × 300 mm, 8 μm, Agilent, UK) were used as columns, and the mobile phase was KH_2_PO_4_ (0.02 M, pH 6.0) with a flow rate of 0.6 mL/min. The molecular weight distribution of the sample was calculated using the GPC software (version A.02.02, Agilent Co., Waldbronn, Germany), and the pullulan polysaccharide standards (molecular mass, 667, 6300, 22,000, 49,700, 216,000, 334,000, and 739,000 Da) were used as molecular mass markers.

### 2.5. Structural Characterization Analysis

#### 2.5.1. Particle Size and ζ-Potential

The particle sizes and ζ-potentials and of RSPs dissolved in distilled water (1 mg/mL) were measured using the dynamic light scattering (DLS) technique on a Malvern Zetasizer, Nano ZS90 at 25 °C. The refractive indices of the dispersed and continuous phases were 1.414 and 1.330, correspondingly. Furthermore, the viscosity was set at 0.8872 cP, and the signal intensity was adjusted to 80 counts per second.

#### 2.5.2. Fourier Transform-Infrared (FT-IR) Spectra

The dried RSP samples (2 mg) were ground with 200 mg of spectroscopic-grade KBr powder and pressed into pellets. Using a Spectrum Two spectrophotometer (PerkinElmer Instruments Co., Shelton, CT, USA), the FT-IR spectrum of every RSP was scanned within the wavenumber range of 4000–450 cm^−1^, with air utilized as the reference medium. Following the spectra collection, the data were evaluated utilizing Spectrum software 10.0 (Spectrum 10™, PerkinElmer, Waltham, MA, USA).

#### 2.5.3. Thermal Stability Properties

The thermal stability properties of RSPs were examined through thermal gravimetric analysis (TGA) using a DTG-60A instrument (Shimadzu Co., Kyoto, Japan). For this analysis, each RSP sample was situated in a platinum crucible, surrounded by a nitrogen atmosphere maintained at a flow rate of 50 mL/min. The crucible was subjected to a controlled heating process at a rate of 10 °C/min, across a temperature spectrum extending from 25 to 800 °C.

#### 2.5.4. X-ray Diffraction (XRD)

The amorphous or crystalline structures of RSPs were assessed through XRD analysis. The experiment was performed with a D8 Advance diffractometer (Bruker, Leipzig, Germany) that had a copper anode emitting Cu Kα radiation. To record XRD spectra, the voltage and current were adjusted to 40 kV and 40 mA. When operating the diffractometer, a scan rate of 10 °/min was used while scanning from 5° to 80°.

#### 2.5.5. Triple-Helix Conformation

The presence of a triple-helix structure within the sample was verified using the Congo red test based on the method established in our prior work [30].

#### 2.5.6. Scanning Electron Microscopy (SEM)

The dehydrated sample was carefully positioned on a holder using adhesive tape on both sides and then coated with gold powder. Next, the SIGMA 300 SEM system (ZEISS, Jena, Germany) was utilized to analyze the surface morphology of RSPs, with an accelerating voltage of 5.0 kV. The magnifications were 50× and 200×.

### 2.6. Assay for Antioxidant Activity

In order to comprehensively assess the antioxidant capabilities of RSPs derived from four different extraction methods, their DPPH, ABTS, and hydroxy radical scavenging activities, as well as their ability to chelate Fe^2+^, were evaluated based on the procedures detailed in our earlier published research [25,30].

### 2.7. Inhibition Assays of α-Glucosidase

#### 2.7.1. Inhibitory Effect on α-Glucosidase

The inhibitory activity of α-glucosidase for each RSP was assessed according to the method described by Chen et al. [31] with slight modifications. To summarize, the process consisted of combining 50 μL of α-glucosidase solution (0.5 U/mL in 0.1 M phosphate buffer, pH 6.8) with 50 μL of RSPs solution (dissolved in 0.1 M phosphate buffer, pH 6.8) at different levels of concentration (0.25, 0.5, 1, 2, 4, and 5 mg/mL). The blend was subsequently placed in an incubator at a temperature of 37 °C for 10 min. Afterward, 50 μL of PNPG (5 mM in 0.1 M phosphate buffer, with a pH of 6.8) was introduced into every vial. Following an additional 20 min period of incubation at 37 °C, the reaction was brought to an end by adding 200 μL of 1 M sodium carbonate solution. The absorbance of the final mixture was measured at 405 nm using a Multiskan SkyHigh microplate reader (Thermo Fisher Scientific Inc., Waltham, MA, USA). Acarbose functioned as a reference standard. The inhibition rate of α-glucosidase was evaluated using the following formula:

(2) $$Inhibitory\;activity\;\left(\%\right)=\left[1-\left(A_{sample}-A_{background}\right)/A_{control}\right]\times 100$$

where subscript sample represents the reaction mixture containing both the samples and α-glycosidase. Subscript background denotes the reaction mixture that excluded the samples. Subscript control indicates the reaction mixture devoid of α-glycosidase. Non-linear regression analysis was used to determine the half-maximal inhibitory concentration (IC_50_) of RSPs, which represent the concentrations required for 50% inhibition in vitro.

#### 2.7.2. Kinetic Characterization of Inhibition

To further elucidate the inhibitory characteristics of RSPs on α-glucosidase, the mechanisms of these inhibitors that interact with the enzyme were investigated by comparing their inhibitory kinetics. Specifically, the inhibitory kinetics models of RSPs against α-glucosidase were determined with escalating concentrations of PNPG (1.0, 2.0, 3.0, and 4.0 mM) as a substrate in the absence and presence of RSPs at 3.0 mg/mL and 5.0 mg/mL. The inhibition type and mechanism exerted by RSPs on α-glucosidase were analyzed by constructing and analyzing Lineweaver–Burk plots using linear regression. From these plots, the Michaelis–Menten constant (*K_m_*) and the maximal velocity (*v_max_*) of the enzymatic reaction were calculated, adhering to the Michaelis–Menten equation [32]:

(3) $$\frac{1}{v}=\frac{1}{v_{\max}}+\frac{K_{m}}{v_{\max}}\cdot \frac{1}{\left[S\right]}$$

#### 2.7.3. Fluorescence Quenching (Fluorescent Spectrometry)

Fluorescence spectroscopy was utilized to analyze the interaction between α-glucosidase and RSPs following a slightly modified method from Jia et al. [33]. In short, various concentrations (0 to 7.0 mg/mL) of RSPs were mixed separately with 2.0 mL of α-glucosidase solution (0.75 mM) in 2.0 mL aliquots and then incubated at 37 °C for 10 min. Preparations of all solutions were made with a phosphate buffer of 0.1 M concentration at a pH of 6.5. Post-incubation, the fluorescence spectra of these mixtures were recorded using a Shimadzu RF-6000 fluorescence spectrophotometer at an excitation wavelength of 280 nm. The background fluorescence was determined by subtracting the buffer readings. Emission signals were collected over a range of 300 to 400 nm, with both excitation and emission slit widths set at 5.0 nm. The maximum fluorescence emission wavelength was used to ascertain the fluorescence quenching parameters and binding constants. Furthermore, the Stern–Volmer equations were utilized to further examine the mechanism of fluorescence reduction, along with the binding constants and sites of RSPs on α-glucosidase [34]:

(4) $$\frac{F_{0}}{F}=1+K_{q}\tau_{0}[Q]=1+K_{SV}[Q]$$

(5) $$\log\frac{F_{0}-F}{F}=\log K_{a}+n\log[Q]$$

where *F*_0_ and *F* represent the fluorescence intensities of α-glucosidase before and after interaction with RSPs, respectively. [*Q*] refers to the concentration of RSPs. The Stern–Volmer quenching constant is denoted as *K*_SV_, and *K*_q_ represents the quenching rate constant. *τ*_0_ indicates the average lifetime of the fluorophore in the absence of the quencher, which is taken as 10^−8^ s. *K*_a_ signifies the binding constant, and *n* is the number of binding sites.

### 2.8. Statistical Analysis

The data were presented as the mean ± standard deviation, derived from three duplicates, unless stated otherwise. The SPSS 27.0 software (SPSS Inc., Chicago, IL, USA) was utilized to perform an analysis of variance (ANOVA). The least significant difference (LSD) test was used to compare multiple means. The threshold for statistical significance was established with a *p*-value of 0.05. The data were graphically represented using OriginPro 2021 Learning Edition (Origin Lab Corp., Northampton, MA, USA).

## 3. Results and Discussion

### 3.1. Extraction Yields and Chemical Compositions

The extraction yields and chemical compositions of RSPs extracted by four different methods are summarized in Table 1. The extraction yields decreased in the order of RSP-A > RSP-W > RSP-S > RSP-C (*p* < 0.05), which indicated that extraction method had a significant impact on polysaccharide yield. The highest extraction yield of RSP-A could be attributed to the alkaline extraction process, which effectively liberated insoluble polysaccharides from cell walls by disrupting hydrogen bonds between cellulose and hemicellulose, thereby enhancing solubility and recovery rates [35]. The observation aligned with the results documented by Chen et al. [16]. RSP-W, with the second highest yield, likely benefited from improved solvent solubility and diffusion, alongside reduced solvent viscosities and solute–matrix interactions in accelerated solvent extraction. Such conditions facilitated more effective moisture penetration into granules, promoting polysaccharide dissolution [36]. Moreover, the lowest extraction yield of RSP-C might stem from the acid solution’s propensity to degrade polysaccharides into free sugars [37]. According to the findings, ASE demonstrated a notable enhancement in RSP yield compared to the methods involving acid and NaCl solutions. This highlighted ASE as an advantageous alternative for extracting polysaccharides from RS fruit. Its benefits included shorter extraction times and increased efficiency under conditions of elevated temperature and pressure.

The total sugar content in RSP-A, RSP-C, RSP-S, and RSP-W was determined to be 77.24 ± 1.31%, 78.23 ± 0.73%, 79.05 ± 0.85%, and 74.76 ± 0.49%, respectively, establishing carbohydrates as the dominant component in these polysaccharides. Protein contents in RSP-A, RSP-C, RSP-S and RSP-W varied slightly (*p* > 0.05), with values of 2.40%, 1.77%, 1.88%, and 1.97%, respectively. The marginally higher protein content in RSP-A could be attributed to the alkaline solution hydrolyzing protein amide bonds [38], aligning with previous findings from blackberry fruit polysaccharides extracted under similar conditions [22]. The content of uronic acid differed significantly (*p* < 0.05) across the extraction methods, with RSP-A showing a notably lower level, possibly due to alkali conditions disrupting the carboxyl groups in uronic acid [39], which was consistent with the previous report [21]. These findings clearly confirmed different extraction methods can influence the yield and chemical composition of RSPs.

### 3.2. Structure Characterizations

#### 3.2.1. Monosaccharide Composition

**Figure 2:**
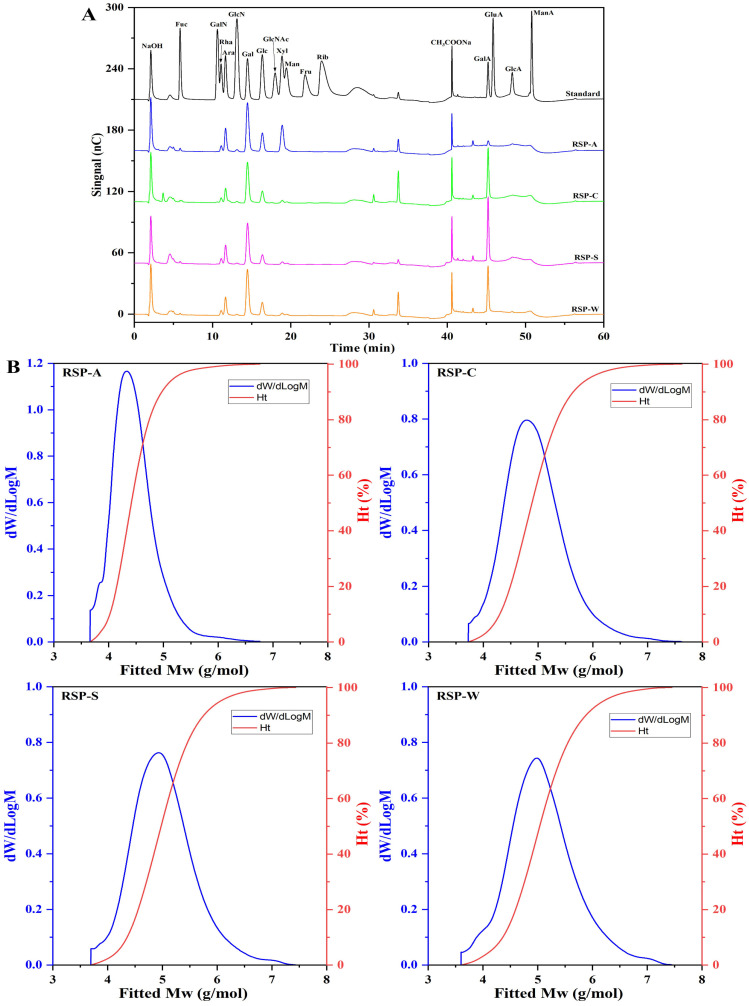
(**A**) Ion chromatograms of standard monosaccharides and RSPs; (**B**) molecular weight distribution curves of RSPs.

As shown in Figure 2A and Table 1, all four RSPs contained the same monosaccharide type of fucose (Fuc), rhamnose (Rha), arabinose (Ara), galactose (Gal), glucose (Glu), xylose (Xyl), mannose (Man), and galacturonic acid (GalA), although their molar ratios differ significantly. RSP-C, RSP-S, and RSP-W predominantly comprised Ara, Gal, and GalA, whereas RSP-A was primarily composed of Ara, Xyl, and Gal. This aligned with prior findings that extraction methods had not significantly changed the monosaccharide types in polysaccharides but did impact their relative proportions. Notably, RSP-A had a significantly higher Xyl content, likely a result of alkaline conditions inducing hydrolysis of polysaccharide chains and disrupting intermolecular hydrogen bonds, thereby influencing monosaccharide profiles [18]. The three RSPs (RSP-C, RSP-S, and RSP-W) had high GalA levels, classifying them as pectic polysaccharides. In contrast, the GalA content of RSP-A was notably lower, echoing the trends observed in uronic acid content, possibly due to the disruptive effect of alkaline conditions on uronic acid’s carboxyl groups [39]. Researchers categorize pectic polysaccharides into two distinct regions: “smooth” and “hairy.” The “smooth” region, specifically the homogalacturonan (HG) domain, comprises partially methylated and acetylated galacturonic acid. In contrast, the “hairy” region encompasses rhamnogalacturonan-I (RG-I), rhamnogalacturonan-II (RG-II), and xylogalacturonan (XGA) domains, characterized by their branched structures [40]. The value of RG-I (%) was calculated as 2Rha (mol%) + Gal (mol%) + Ara (mol%), and the RG-I (%) value of the three RSPs exceeded 40%, indicating that the three RSP types were rich in Ara or Gal side chains, and RSP-W was the highest, reaching 55.48%. Generally, a higher galacturonic acid content was associated with enhanced biological activity in polysaccharides [41]. Therefore, these variations in monosaccharide compositions could affect the biological functions of the RSPs.

#### 3.2.2. Molecular Weight Distribution

As shown in the molecular weight distribution curves (Figure 2B), all four RSPs displayed a signal peak, indicating their high purity. In general, the distribution of molecular weight in polysaccharides was assessed by considering the number-average molecular weight (Mn), weight-average molecular weight (Mw), and the polydispersity index (Mw/Mn). The corresponding molecular weight parameters for RSPs are detailed in Table 1. Remarkably, RSP-W had the highest Mw at 416.79 kDa, whereas RSP-A had the lowest at 60.98 kDa. This disparity suggested that under high extraction temperature, RSPs could tend to aggregation, and their chains may be fragmented during alkaline extraction, consistent with findings from previous studies [18,26]. Mw is a crucial determinant of polysaccharide solubility. The lower Mw of RSP-A indicated its better water solubility at room temperature. Polysaccharides with smaller polydispersity indices, which indicate a narrower distribution of molecular weights, are associated with higher levels of homogeneity [42]. According to Table 1, the polydispersity indices for RSP-A, RSP-C, RSP-S, and RSP-W were 2.91, 6.16, 6.27, and 7.79, respectively. These values indicated that all the RSP samples were characterized by a broad range of molecular weights, suggesting their polydispersity as polymers. The highest polydispersity observed in RSP-W indicated a considerable presence of small Mw fragments. On the other hand, the molecular weight distribution of RSP-A was the most uniform, which could be attributed to the ability of the alkali to depolymerize the RSP or disturb noncovalent bonds between molecules, aligning with its observed low Mw. These results implied that different extraction methods could significantly influence the molecular weight and size distribution of RSPs.

#### 3.2.3. FT-IR Analysis

**Figure 3:**
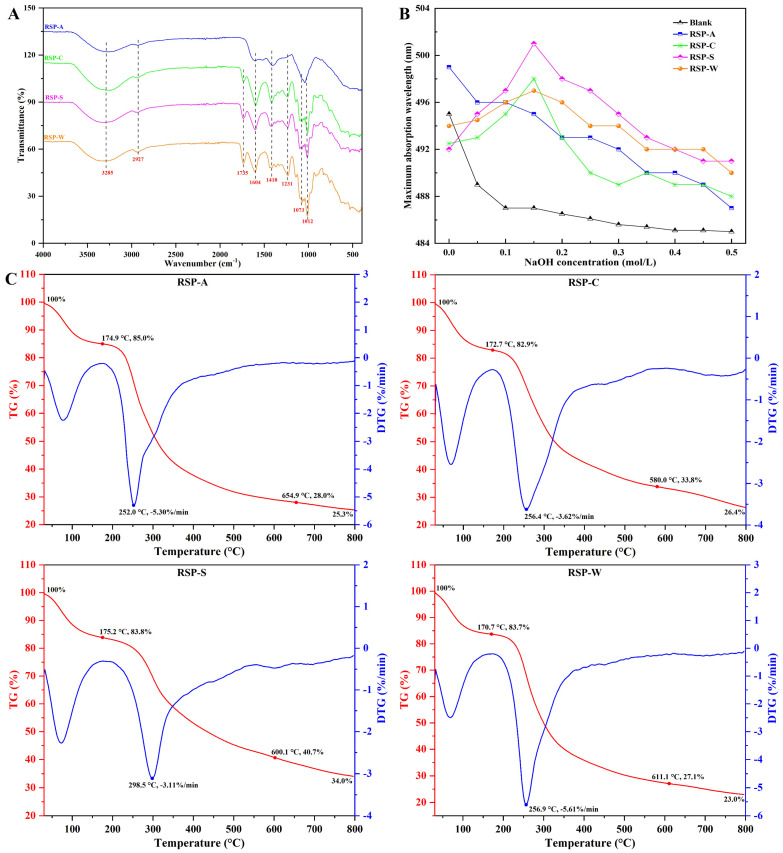
(**A**) FT-IR spectra of RSPs; (**B**) effect of RSPs on the absorbance of Congo red at various NaOH concentrations; (**C**) TG and DTG curves of RSPs.

The characteristic FT-IR spectra of the four RSPs are presented in Figure 3A. The O–H stretching vibration was indicated by the broad and intense broadbands within the range of 3600–3200 cm^−1^, whereas the C–H stretching vibrations of the CH_2_ groups were attributed to the weak bands at 2927 cm^−1^ [43]. The presence of uronic acid in all RSP samples was confirmed by identifying absorption peaks at approximately 1735 cm^−1^ and 1604 cm^−1^, corresponding to the stretching vibrations of esterified and free carboxyl functional groups (-COOR), respectively [20]. The degree of esterification can be quantified by calculating the ratio of the peak area at 1735 cm^−1^ to the combined areas of both peaks [44]. It was evident that RSP-C had a low degree of esterification compared to RSP-S and RSP-W because of the desertification under acid extraction conditions. Specifically, the 1604 cm^−1^ peak in RSP-A was significantly weaker compared to the other samples, illustrating a reduced uronic acid content in RSP-A. This observation was in line with the results from monosaccharide composition and uronic acid content analyses. Moreover, the absorption peak of RSP-S and RSP-C at 1735 cm^−1^ was significantly weaker than that of RSP-W, reflecting more extensive cleavage of ester bonds and release of free carboxyl groups in RSP-S and RSP-C [45]. The absence of this peak in RSP-A may correlate with its significantly low uronic acid content. Additionally, the peaks at 1073 and 1012 cm^−1^ were likely indicative of the pyranose sugar form [46]. These FT-IR spectra demonstrated that while extraction methods significantly altered the monosaccharide composition of RSPs, they did not affect the fundamental distribution of sugar rings and glycosidic bonds.

#### 3.2.4. Triple-Helix Analysis

Congo red’s interaction with polysaccharides that possess a triple-helix structure results in a stable complex whose maximum absorption wavelength (λmax) increases in weakly alkaline solutions and decreases in strongly alkaline ones. This characteristic makes Congo red an effective tool for identifying triple-helical structure in polysaccharides. Figure 3B illustrates that, as the concentration of NaOH increased, a noticeable red shift of the λmax occurred in RSP-C, RSP-S, and RSP-W compared to the pure Congo red solution. This shift indicated the presence of a triple-helical conformation in these polysaccharides. In contrast, for RSP-A, an increase in alkaline concentration from 0 to 0.5 M resulted in a decrease in λmax, implying the absence of a triple-helical structure in RSP-A. These observations revealed that the extraction method may influence the conformation of polysaccharides, corroborating the finding from previous study [22]. The underlying reason for this could be due to a relationship between the molecular weight of polysaccharides and their ability to form helical structure [47].

#### 3.2.5. Thermal Characteristic

Thermogravimetric stability is a particularly important physicochemical property of polysaccharides in the food industry, which reflects their mass variations over time and temperature. Figure 3C presents the thermogravimetric (TG) and differential thermogravimetric (DTG) profiles of RSPs across a temperature range of 30 to 800 °C. This degradation occurred in three distinct stages, as summarized in Table 2. Initially, mass loss primarily resulted from the vaporization of both free and attached water in RSPs [48]. Variations in water loss among RSP samples suggested their different water contents. Specifically, RSP-C, RSP-S, and RSP-W exhibited a markedly greater mass decrease during this evaporation process compared to RSP-A. The next stage involved the breakdown of glycosidic bonds and polysaccharide side chains [49]. During this stage, RSP-C and RSP-S showed a significantly lower peak decomposition rate (3.62 and 3.11%/min, respectively), suggesting their better thermal stability than other RSP samples. The final stage was marked by the fragmentation of stronger structures, including the cleavage of C–O bonds and glucosidic groups, leading to the disintegration of the polysaccharide backbone [50]. Here, RSP-C’s mass loss exceeded that of RSP-A, RSP-S, and RSP-W. Furthermore, the total mass losses of them were differed in the following order: RSP-W > RSP-A > RSP-C > RSP-S. The total mass loss value of RSP-S was significantly lower than that of the other three RSP samples. Overall, the above results revealed that RSP-S had the greatest thermal stability. Moreover, the observations underscored that the different extraction methods could affect the thermal stability of RSP, potentially attributable to their distinct physicochemical characteristics identified earlier.

#### 3.2.6. Particle Sizes and ζ-Potentials Analysis

**Figure 4:**
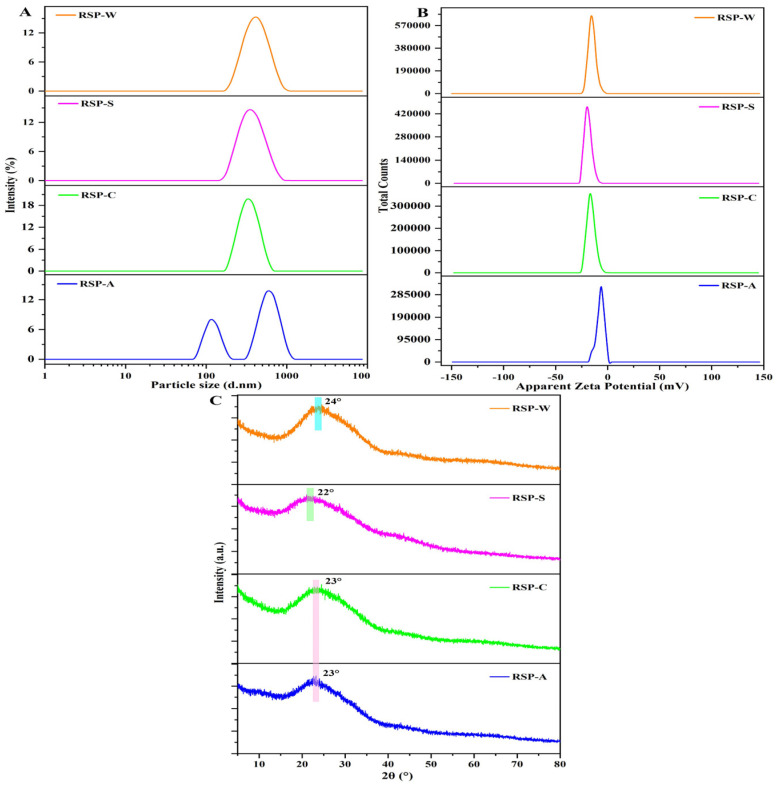
(**A**) Particle size distribution curves of RSPs; (**B**) ζ-potential distribution curves of RSPs; (**C**) XRD spectra of RSPs.

The stability of polysaccharides solution could be inferred from the behavior of hydrodynamic particles and the charge [51]. Particle size, reflecting the aggregation level of polysaccharide molecules, serves as an indirect metric for molecular weight to a certain extent [22]. Figure 4A,B displays the particle size and ζ-potential distribution for the four RSP samples, with average values detailed in Table 3. As evidenced in Figure 4A, all four RSP solutions showed a broad range in particle size distribution, aligning with molecular weight distribution findings. According to Table 3, RSP-C and RSP-S exhibited similar average particle sizes, while RSP-A stood out with the smallest size at 322 nm, likely due to its lower molecular weight. Consequently, RSP-A appeared to have more uniform particles and greater stability in aqueous solution compared to the other three RSPs. All four RSPs possessed negative charge, yet their ζ-potential values did not reach the |±30| mV threshold, varying between 5.6 and 18.3. According to Table 3, RSP-S exhibited the greatest negative charge, suggesting that its intermolecular repulsion surpassed that of other RSP variants, possibly attributable to its elevated uronic acid content. On the other hand, the lowest level of RSP-A’s absolute potential may be closely associated with a higher concentration of neutral sugar, resulting in limited electrical conductivity. Generally, particles with a ζ-potential less than |±30| mV had not enough repulsive force to prevent aggregation [52]. Hence, this meant all the RSP particles tended to aggregate in the solution.

#### 3.2.7. Crystal Structure Analysis

The structural nature of the RSPs, whether amorphous or crystalline, was further investigated through XRD analysis. As illustrated in Figure 4C, each RSP sample exhibited a singular, subdued bread-like peak around 2θ ≈ 23°, suggesting all RSPs possessed a semi-crystalline structure. However, variations in the intensity of these diffraction peaks across the different RSPs highlighted differences in their microcrystalline structures. These variations, when considered alongside Mw distribution, FT-IR, and Congo red assay results, indicated that the structural characteristics of RSPs were significantly influenced by their extraction methods.

#### 3.2.8. SEM Analysis

**Figure 5:**
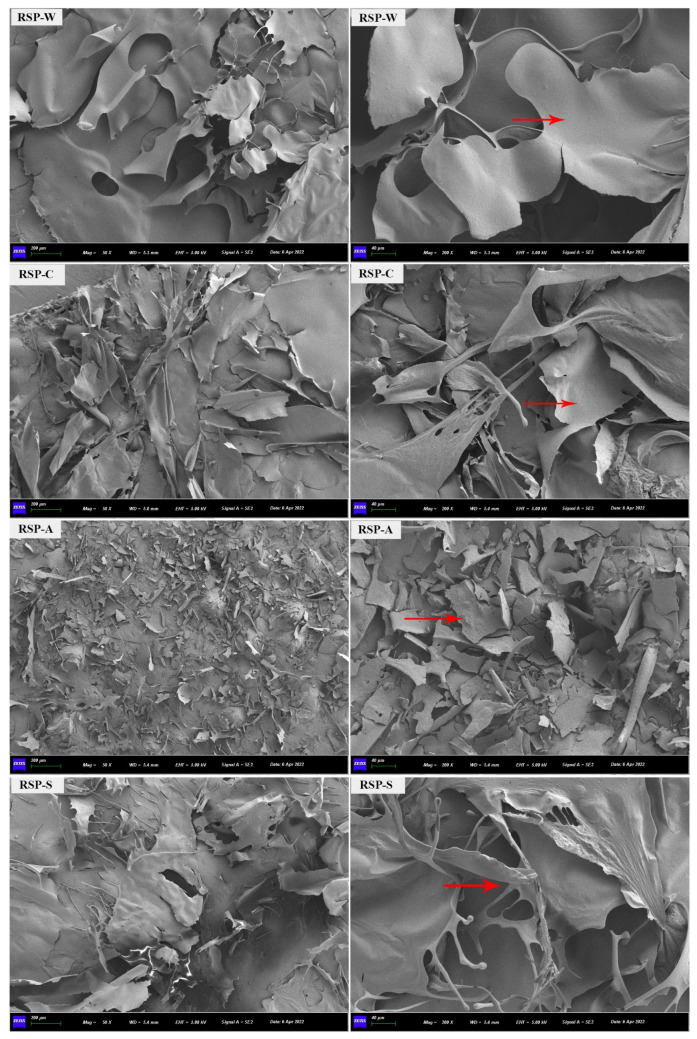
Scanning electron micrographs of RSPs.

The four RSP samples are depicted in Figure 5, displaying noticeable differences in size and shape in their distinct surface microstructures. RSP-W and RSP-S appeared to have a tightly packed, smooth yet irregular surface with a prominent flaky structure. The aggregation of polysaccharide molecules was responsible for the larger particle size observed in the SEM images of both RSP-W and RSP-S. Contrastingly, RSP-C featured a greater abundance of smaller fragments compared to RSP-W, likely stemming from the cross-linked aggregation of galacturonic acid. Meanwhile, RSP-A presented a relatively dense structure with an irregular shape and a surface texture akin to an atypical brick pattern. Its particles were smaller and more uniformly distributed than those in the other three RSP samples. This uniformity suggested that RSP-A underwent degradation during alkali extraction, possibly related to its lower uronic acid content, resulting in fewer interaction points among particles [53]. The morphologies and microstructures observed supported the findings from the well in line with the results obtained from the analyses of molecular weight and particle size distribution.

### 3.3. In Vitro Antioxidants Analysis

**Figure 6:**
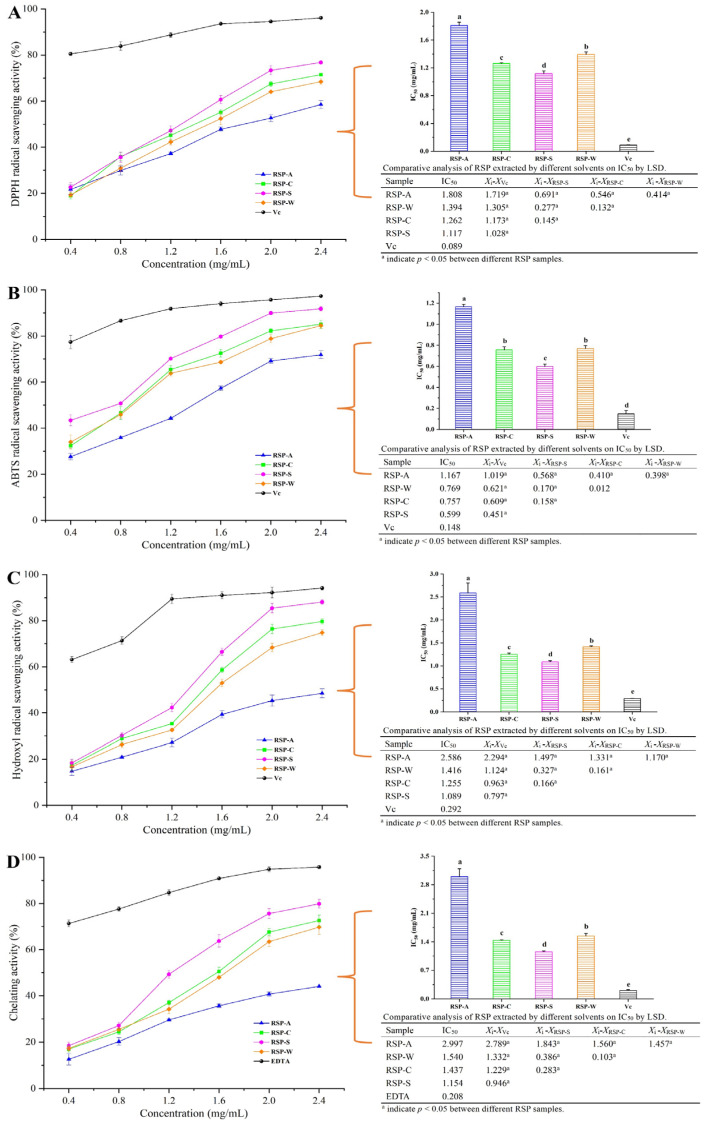
Antioxidant activities of PSPs. (**A**) DPPH radical scavenging activity; (**B**) ABTS radical scavenging activity; (**C**) hydroxyl radical scavenging activity; (**D**) chelating activity on ferrous ion. Values for IC_50_ with no letters in common are significantly different (*p* < 0.05).

The development of various chronic illnesses, such as heart disease, degenerative brain conditions, cancer, diabetes, and aging, is significantly influenced by oxidative stress [54]. The ability of plant polysaccharides to effectively counteract oxidative stress has gained recognition [55]. To evaluate the antioxidant potential of plant-derived polysaccharides, analytical models such as DPPH, ABTS, OH radical scavenging, and ferrous ion chelation were widely used [26]. In this study, these methods were utilized to evaluate the antioxidant capabilities of RSPs extracted through four different methods (Figure 6).

The four RSPs exhibited a quadratic concentration-dependent pattern (*p* < 0.05) in terms of their demonstrated DPPH radical scavenging abilities (*y*), as observed in Figure 6A. The corresponding linear equations were as follows: *y* = −2.43*x*² + 26.53*x* + 9.87 (*R*² = 0.968) for RSP-A; *y* = −6.48*x*² + 44.33*x* + 2.59 (*R*² = 0.991) for RSP-C; *y* = −5.94*x*² + 44.23*x* + 4.97 (*R*² = 0.993) for RSP-S; and *y* = −5.11*x*² + 40.61*x* + 3.19 (*R*² = 0.994) for RSP-W. The IC_50_ values were 1.808 mg/mL for RSP-A, 1.262 mg/mL for RSP-C, 1.117 mg/mL for RSP-S, and 1.394 mg/mL for RSP-W, indicating that RSP-S exhibited the highest DPPH radical scavenging activity, followed by RSP-C, RSP-W, and RSP-A. All RSPs displayed ABTS radical-scavenging activity that increased with concentration (0.4–2.4 mg/mL). The scavenging activities (*y*) exhibited a quadratic trend in relation to the concentration of polysaccharides (x) (*p* < 0.05). The following linear equations were observed: *y* = 2.97*x*² + 16.87*x* + 20.04 (*R*² = 0.964) for RSP-A; *y* = −10.62*x*² + 56.47*x* + 11.37 (*R*² = 0.995) for RSP-C; *y* = −14.10*x*² + 70.49*x* + 4.27 (*R*² = 0.979) for RSP-S; and *y* = −5.52*x*² + 40.30*x* + 19.23 (*R*² = 0.952) for RSP-W. RSP-A exhibited an IC_50_ value of 1.167 mg/mL for ABTS radical scavenging, while RSP-C, RSP-S, and RSP-W showed IC_50_ values of 0.757 mg/mL, 0.599 mg/mL, and 0.769 mg/mL, respectively. The findings revealed that RSP-S displayed the highest capacity for ABTS radical scavenging, achieving a maximum scavenging rate of 91.83%, followed by RSP-C (85.14%), RSP-W (84.51%), and RSP-A (71.85%).

The scavenging ability of RSPs for the hydroxyl radical (*y*) showed a quadratic correlation with the concentration (*x*) (*p* < 0.05). The respective quadratic equations were as follows: for RSP-A, *y* = −1.63*x*² + 23.36*x* + 3.54 (*R*² = 0.970); for RSP-C, *y* = 3.77*x*² + 24.60*x* + 2.11 (*R*² = 0.915); for RSP-S, *y* = −3.34*x*² + 46.80*x* − 3.39 (*R*² = 0.971); and for RSP-W, *y* = 5.89*x*² + 14.82*x* + 8.11 (*R*² = 0.942). At a concentration of 2.4 mg/mL, the scavenging abilities for RSP-A, RSP-C, RSP-S, and RSP-W were 48.49%, 79.70%, 88.11%, and 74.82%, respectively. This demonstrated that RSPs, especially RSP-S with the highest scavenging rate and lowest IC_50_ value (1.089 mg/mL), had significant hydroxyl radical scavenging activity. Ferrous ion chelating activities of RSPs, presented in Figure 6D, also followed a quadratic concentration-dependent pattern (*p* < 0.05). The quadratic equations for RSP-A, RSP-C, RSP-S, and RSP-W were *y* = −1.63*x*^2^ + 23.36*x* + 3.54 (*R*^2^ = 0.970), *y* = 3.77*x*^2^ + 24.58*x* + 2.12 (*R*^2^ = 0.915), *y* = −3.34*x*^2^ + 46.80*x* − 3.39 (*R*^2^ = 0.971), and *y* = 5.89*x*^2^ + 14.82*x* + 28.11 (*R*^2^ = 0.942), respectively. Among these, RSP-S exhibited a significantly better chelating ability than RSP-A, RSP-C, and RSP-W across all tested concentrations (0.2 to 2.4 mg/mL). The IC_50_ values for RSPs were in the order of RSP-S (1.154 mg/mL) < RSP-C (1.437 mg/mL) < RSP-W (1.540 mg/mL) < RSP-A (2.997 mg/mL), all of which were significantly greater than that of EDTA (0.208 mg/mL, *p* < 0.01). This suggested that although RSPs exhibit a relatively inferior chelating capability in comparison to EDTA, their capacity remained significantly robust.

According to previous reports, the uronic acid content, molecular weight, monosaccharide composition, and chain conformation were found to have an impact on the antioxidant activity of natural polysaccharides [18,56]. Polysaccharides generally exhibited radical scavenging activity due to their ability to donate electrons or hydrogen [57]. The uronic acid groups within polysaccharides can engage with the hydrogen atom on the anomeric carbon, enhancing their antioxidant capacity [58]. Moreover, reactive groups like ketones or aldehydes, particularly in acidic environments, enhance the liberation of hydrogen from O-H bonds, reinforcing the efficacy of antioxidants [59]. As a result, polysaccharides rich in uronic acid often display potent antioxidant activity. Lower molecular weight polysaccharides, having more readily available hydroxyl groups, can effectively neutralize free radicals, translating to increased antioxidant activity [48]. The monosaccharide composition also plays a significant role, as it interacts with different cellular membrane receptors and influences intracellular signaling pathways [60]. It was suggested that polysaccharides with lower glucose content may exhibit stronger antioxidant properties [61]. Furthermore, the polysaccharide chain conformation, like the triple-helix structure, is important in maintaining interactions with free radicals [62]. Given the distinct physicochemical properties of the four RSP types, it can be inferred that RSP-S’s higher uronic acid content, a greater proportion of beneficial monosaccharides (lower glucose content), and relatively lower molecular weight could contribute positively to its superior antioxidant capacity. However, the minimal antioxidant capacity of RSP-A, obtained through the use of an alkaline solution, might stem from the breakdown of carboxyl groups in uronic acid and the deterioration of the triple-helical arrangement of the polysaccharide during the rigorous extraction process.

### 3.4. α-Glucosidase Inhibition Activity Assay

**Figure 7:**
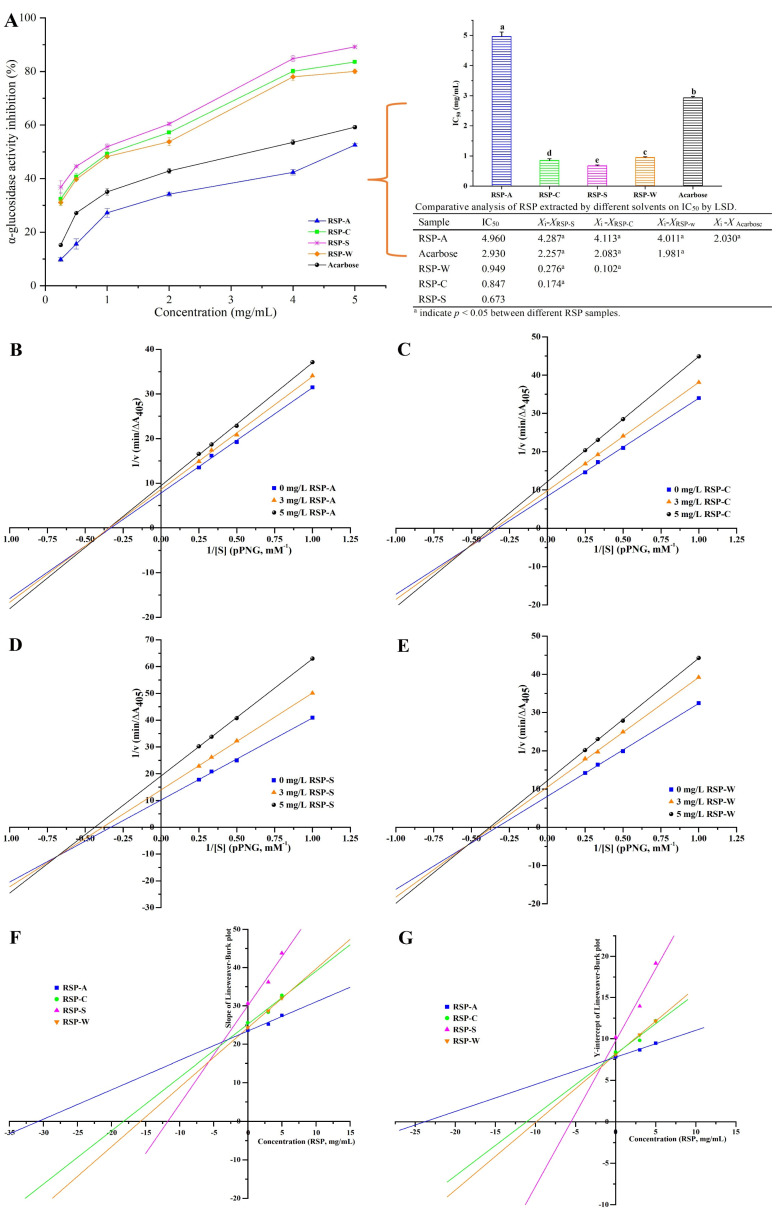
Inhibitory effect of RSPs on α-glucosidase activity (**A**); Lineweaver–Burk plots for different concentrations of RSP-A (**B**), RSP-C (**C**), RSP-S (**D**), and RSP-W (**E**) against α-glucosidase activity; the slope of the Lineweaver–Burk plot versus concentration for RSPs (**F**); the Y-intercept of the Lineweaver–Burk plot versus concentration for RSPs (**G**). Values for IC_50_ with no letters in common are significantly different (*p* < 0.05).

Figure 7A demonstrates the dose-dependent inhibitory effects of RSPs and acarbose on α-glucosidase activity. At a concentration of 5.0 mg/mL, the α-glucosidase inhibition rates for RSP-A, RSP-C, RSP-S, and RSP-W were 52.54%, 83.53%, 89.19%, and 80.15%, respectively, with RSP-C, RSP-S, and RSP-W showing higher efficacy than acarbose (59.26%). The IC_50_ values for the inhibition of α-glucosidase activity by RSP-A, RSP-C, RSP-S, and RSP-W were 4.96, 0.847, 0.673, and 0.949 mg/mL, respectively. This indicated that RSP-S had the most potent inhibitory activity among the four RSPs (*p* < 0.05), and the IC_50_ values for RSP-C, RSP-S, and RSP-W were considerably lower than that of acarbose (2.93 mg/mL) (*p* < 0.05). These results revealed the potential of RSPs, particularly RSP-S, as natural α-glucosidase inhibitors for medical and food industry applications. Prior research indicated that the -OH and -COOH groups on polysaccharide branched chains can form strong hydrogen bonds with α-glucosidase residues, inhibiting the enzyme’s activity [19,63]. Polysaccharides with a higher uronic acid content and lower molecular weight are deemed more effective due to increased exposure to active sites [64,65]. Furthermore, the glucose content and triple-helical structure of polysaccharides were also contributing factors to their α-glucosidase inhibitory capability [66,67]. Considering the chemical and structural properties of RSPs, the exceptional inhibitory activity of RSP-S could be attributed to its relatively smaller molecular weight, higher uronic acid content, and more extended chains, enhancing its interaction with the enzyme’s active sites. Thus, further studies into the inhibitory kinetics and mechanism of RSPs on α-glucosidase were carried out, potentially providing a theoretical foundation for using RSPs as a promising candidate in medical and food industry applications targeting α-glucosidase.

### 3.5. Inhibitory Kinetics Analysis

The inhibition type of RSPs on α-glucosidase was investigated through the Lineweaver–Burk double reciprocal plot and the Michaelis–Menten equation. Figure 7B–E depicts that for each RSP, three plot lines intersected in the third quadrant of the coordinate axis. Moreover, with increasing concentrations of inhibitors, both *V*_max_ and *K*_m_ of three plots for each RSP decreased, as detailed in Table 4. It is manifested that all four RSPs exhibited a mixed-type inhibition mechanism on α-glucosidase. This observation was in line with similar studies, such as the mixed-type inhibitory effects of *R. roxburghii* Tratt fruit polysaccharide on α-glucosidase [68].

To determine the enzyme–inhibitor dissociation constant (*K*_i_), one can calculate the absolute value of the X-intercept in a fitted curve obtained by analyzing the correlation between the slope of the Lineweaver–Burk plot and the concentration of the inhibitor being tested [69]. The *K*_is_ value, which represents the dissociation constant of the enzyme–substrate–inhibitor complex, can be determined by calculating the absolute value of the X-intercept of the fitted curve using the Y-intercept of the Lineweaver–Burk plot and the concentration of the inhibitor [5]. Figure 7F,G and Table 4 revealed the calculated *K*_i_ values for RSP-A, RSP-C, RSP-S, and RSP-W as 30.746, 18.183, 11.727, and 15.731 mg/mL, respectively. The corresponding *K*_is_ values were 23.881, 11.12, 5.565, and 9.889 mg/mL. Generally, lower *K*_i_ and *K*_is_ values indicate stronger binding in both the inhibitor–enzyme complexes and the inhibitor–enzyme–substrate complexes, as they represent less dissociation of these complexes [70]. Thus, RSP-S, with the smallest *K*_i_ and *K*_is_ values, demonstrated a stronger affinity for α-glucosidase compared to the other RSPs. This observation aligned with the results of the IC_50_ values, further validating RSP-S’s potent inhibitory activity.

### 3.6. Fluorescence Quenching Analysis

**Figure 8:**
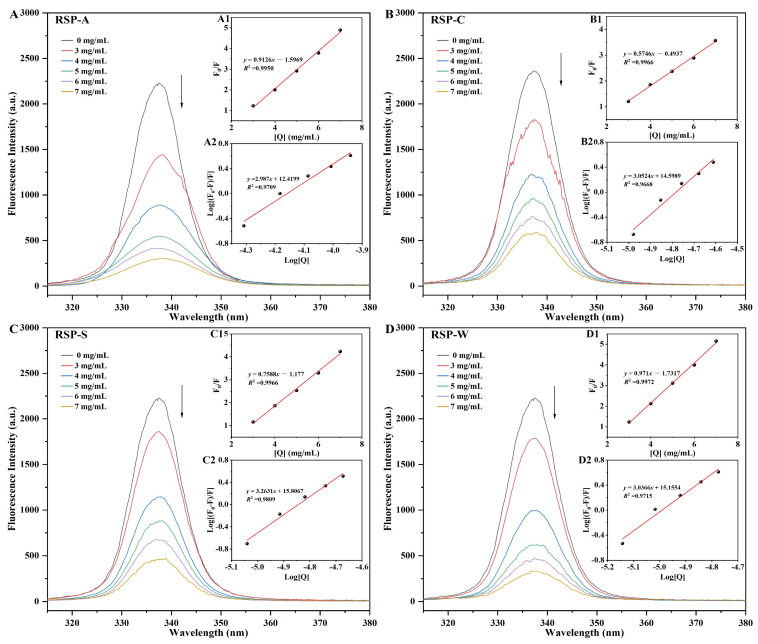
(**A**–**D**) Fluorescence spectra of α-glucosidase in the presence of RSPs with different concentrations; the Stern–Volmer plots of α-glucosidase with different concentrations of RSP-A (**A1**), RSP-C (**B1**), RSP-S (**C1**), and RSP-W (**D1**); the double logarithm regression plots of log [(F_0_ − F)/F] against log [Q] for α-glucosidase with different concentrations of RSP-A (**A2**), RSP-C (**B2**), RSP-S (**C2**), and RSP-W (**D2**).

Fluorescence quenching pertains to a process where the fluorescence intensity of a substance is reduced due to physical or chemical interactions with other molecules [71]. In proteins, fluorescence predominantly arises from three aromatic amino acids—tyrosine, phenylalanine, and tryptophan, with tryptophan being the major contributor to fluorescence emission. Consequently, changes in the inherent fluorescence of glycosidases, which contain tryptophan residues, can effectively indicate alterations in both the protein and its surrounding environment [72]. To further explore the interaction mechanism between RSPs and α-glucosidase, fluorescence quenching experiments were carried out, with results depicted in Figure 8A–D. The fluorescence intensity decreased as the concentrations of the four RSP types increased, indicating that all RSPs could quench the natural fluorescence of α-glucosidase and bind to the enzyme. Significantly, a slight red shift in the peak absorption wavelength (λmax) of α-glucosidase was observed as RSP concentrations increased. For example, in the case of RSP-C (Figure 8B), the λmax shifted from 337.4 nm to 338.4 nm, and for RSP-S (Figure 8C), from 337.4 nm to 338.9 nm. These red shifts suggested the formation of a hydrophobic microenvironment around the tryptophan residues within α-glucosidase, leading to the exposure of these residues and indicating an unfolding of the protein structure [73]. Similar red shifts were also observed in the interaction of α-glucosidase with RSP-A (from 337.4 nm to 338.2 nm, Figure 8A) and RSP-W (from 337.4 nm to 338.1 nm, Figure 8D), reinforcing the consistent effect of RSPs on the protein structure of α-glucosidase.

To explicate the interaction mechanism behind the fluorescence quenching of α-glucosidase by RSPs, the Stern–Volmer equation was employed at 310 K. By examining the Stern–Volmer quenching constant value (*K_SV_*), this equation differentiates between static and dynamic quenching. The formation of a non-luminous compound with minimal or absent light emission leads to static quenching, whereas dynamic quenching takes place when the quenching agent collides with the fluorescent group, resulting in energy transfer [33,74]. Figure 8(A1–D1) shows the Stern–Volmer plots for the RSPs–α-glucosidase interaction, all exhibiting a linear fit with high regression coefficients (*R*² > 0.99). The *K_SV_* values calculated for RSP-A, RSP-C, RSP-S, and RSP-W were 5.57 × 10^4^, 1.64 × 10^5^, 2.50 × 10^5^, and 4.05 × 10^5^ M^−1^, respectively. Additionally, the quenching rate constants (*K_q_*) were determined to be 5.57 × 10^12^, 1.64 × 10^13^, 2.50 × 10^13^, and 4.05 × 10^13^ M^−1^ s^−1^ (Table 4), substantially higher than the maximum dynamic quenching constant of 4 × 10^10^ M^−1^ s^−1^ [75]. This manifested that the predominant interaction mechanism between RSPs and α-glucosidase was static quenching. The binding sites and equilibrium between free and bound molecules were further analyzed using Equation (5). The *n* values, derived from Figure 8(C2–D2) for all RSP types, were approximately 3, implying multiple binding sites on α-glucosidase. The association constant (*K_a_*), indicative of the formation efficiency of the inhibitor-α-glucosidase complex [76], varied among the four RSPs. Specifically, the *K_a_* values for RSP-A, RSP-C, RSP-S, and RSP-W were 2.63 × 10^12^, 3.97 × 10^14^, 6.41 × 10^15^, and 1.43 × 10^15^ M^−1^, respectively. This highlighted that RSP-S exhibited the highest binding efficiency, pointing to a more effective interaction with α-glucosidase.

## 4. Conclusions

Functional polysaccharides were obtained from seedless chestnut rose fruit in the current investigation through the utilization of four distinct techniques, which encompassed accelerated solvent extraction, acid extraction, alkali extraction, and NaCl solution extraction. Significant differences were observed among the four types of RSP in terms of uronic acid content, molecular weight, particle size distribution, monosaccharide composition, triple-helix structure, thermal stability, surface morphology, antioxidant activity, and hypoglycemic activity. However, they exhibited similar infrared spectra and crystalline characteristics. Specifically, RSP-A exhibited the highest extraction yield and xylose content, along with the lowest molecular weight. RSP-W had the highest molecular weight, while RSP-S contained the highest amount of uronic acid and showed the greatest thermal stability and a distinct triple-helix structure. RSP-S may have exhibited superior antioxidant and α-glucosidase inhibitory properties compared to the other three samples because of its elevated uronic acid level, advantageous monosaccharide composition, and comparatively lower molecular weight. Enzyme kinetic analysis indicated that all four RSP types inhibited α-glucosidase through a mixed-type mechanism. Additionally, RSP-S showed the highest binding efficiency with α-glucosidase, with all RSPs quenching the fluorescence of α-glucosidase mainly through static interaction and binding to three distinct sites.

The extraction with NaCl solution appeared particularly effective in yielding polysaccharides from seedless chestnut rose fruit with robust antioxidant and hypoglycemic activities. RSP-W also showed significant performance in these domains, albeit slightly lower than RSP-S. Our research indicated that the ASE technique held promise as a viable option for extracting RSPs due to its high productivity and noteworthy efficacy, offering a convenient approach for industrialization. These findings may lay a theoretical foundation for the design, processing, and application of RSPs as functional ingredients or natural antioxidants/hypoglycemic agents in the food and pharmaceutical industries through various extraction methods. However, further research is needed to explore the detailed structure–activity relationships and in vivo activities of RSPs.

## Figures and Tables

**Table 1:** Extraction yield, chemical composition, sugar composition, and molecular weight distribution of polysaccharides from seedless chestnut rose fruit extracted by different methods.

	RSP-A	RSP-C	RSP-S	RSP-W
Yield (%)	7.11 ± 0.75 ^a^	4.36 ± 0.33 ^d^	5.04 ± 0.16 ^c^	5.95 ± 0.30 ^b^
Chemical composition (%, *w*/*w*)				
Total sugar	77.24 ± 1.31 ^ab^	78.23 ± 0.73 ^a^	79.05 ± 0.85 ^a^	74.76 ± 0.49 ^b^
Protein	2.40 ± 0.11 ^a^	1.77 ± 0.08 ^a^	1.88 ± 0.02 ^a^	1.97 ± 0.03 ^a^
Uronic acid	3.83 ± 0.13 ^d^	32.31 ± 0.92 ^b^	37.67 ± 0.34 ^a^	28.10 ± 0.53 ^c^
Monosaccharide composition (molar ratio, %)				
Fucose	1.12	1.19	0.43	0.36
Rhamnose	4.93	4.38	4.41	4.14
Arabinose	14.2	9.89	10.75	11.05
Galactose	38.56	32.14	28.73	36.15
Glucose	11.47	7.22	5.1	7.93
Xylose	23.81	2.15	1.92	2.09
Mannose	1.49	1.24	1.02	1.17
Galacturonic acid	4.42	41.79	47.64	37.11
HG/%	—	37.41	43.23	32.97
RG-I/%	—	50.79	48.30	55.48
Molecular weight distribution				
Mw (kDa)	60.98 ± 2.31 ^d^	285.62 ± 6.93 ^c^	392.42 ± 4.57 ^b^	416.79 ± 12.22 ^a^
Mn (kDa)	20.93 ± 0.95 ^c^	46.34 ± 0.66 ^b^	52.56 ± 1.04 ^a^	53.54 ± 0.74 ^a^
Mw/Mn	2.91 ± 0.10 ^c^	6.16 ± 0.35 ^b^	6.27 ± 0.29 ^b^	7.79 ± 0.27 ^a^

HG/% = Galacturonic acid − Rhamnose. RG-I/% = 2 Rhamnose + Galactose + Arabinose. “—” means that RSP-A cannot be characterized by this value. Values with different letters in the same row indicate significant differences (*p* < 0.05).

**Table 2:** Thermogravimetric stabilities of polysaccharides from seedless chestnut rose fruit extracted by different methods.

Sample	Stage 1	Stage 2				Stage 3		Total Mass Loss (%)
	Mass Loss (%)	Start Temperature (°C)	Maximum Decomposition Rate (%/min)	*T_max_* (°C)	Mass Loss (%)	Start Temperature (°C)	Mass Loss (%)	
RSP-A	15.0 ± 0.7 ^b^	174.9 ± 5.5 ^a^	5.30 ± 0.33 ^a^	252.0 ± 6.3 ^b^	57.0 ± 1.4 ^a^	654.9 ± 4.8 ^a^	2.7 ± 0.2 ^c^	74.7 ± 2.4 ^a^
RSP-C	17.1 ± 0.8 ^a^	172.7 ± 6.1 ^a^	3.62 ± 0.20 ^b^	256.4 ± 7.1 ^b^	49.1 ± 2.0 ^b^	580.0 ± 5.3 ^c^	7.4 ± 0.5 ^a^	73.6 ± 2.1 ^a^
RSP-S	16.2 ± 0.6 ^a^	175.2 ± 4.9 ^a^	3.11 ± 0.35 ^b^	298.5 ± 7.9 ^a^	43.1 ± 1.6 ^c^	600.1 ± 6.1 ^b^	5.3 ± 0.4 ^b^	66.0 ± 0.9 ^b^
RSP-W	16.3 ± 0.6 ^a^	170.7 ± 5.0 ^a^	5.61 ± 0.54 ^a^	256.9 ± 5.2 ^b^	56.6 ± 2.1 ^a^	611.1 ± 5.9 ^b^	4.1 ± 0.4 ^b^	77.0 ± 1.2 ^a^

Values are presented as mean ± SD (*n* = 3). Values in the same column with different letters indicate significant differences (*p* < 0.05).

**Table 3:** Particle size and ζ-potential of polysaccharides from seedless chestnut rose fruit extracted by different methods.

Samples	RSP-A	RSP-C	RSP-S	RSP-W
Particle size (nm)	322.0 ± 11.7 ^c^	353.6 ± 12.5 ^b^	361.0 ± 9.8 ^b^	400.2 ± 5.0 ^a^
PDI	0.475	0.318	0.366	0.493
ζ-potential (mV)	−5.6 ± 0.2 ^c^	−16.0 ± 0.1 ^b^	−18.3 ± 0.7 ^a^	−15.6 ± 0.6 ^b^

^a–c^ Values with no letters in common are significantly different (*p* < 0.05).

**Table 4:** Kinetic parameters of *α*-glucosidase inhibition in the presence of polysaccharides from seedless chestnut rose fruit extracted by different methods.

	RSP-A (mg/mL)			RSP-C (mg/mL)			RSP-S (mg/mL)			RSP-W (mg/mL)		
	Control	3	5	Control	3	5	Control	3	5	Control	3	5
inhibition type	mixed inhibition			mixed inhibition			mixed inhibition			mixed inhibition		
*K_m_* (mM)	3.023	2.930	2.903	3.072	2.893	2.691	3.004	2.683	2.286	3.013	2.744	2.634
*V_max_* (∆A405/min)	0.127	0.116	0.106	0.120	0.102	0.0822	0.098	0.0741	0.0522	0.124	0.0955	0.0823
*K_i_* (mg/mL)	30.746			18.183			11.727			15.731		
*K_is_* (mg/mL)	23.881			11.120			5.565			9.889		
*K_SV_* (M^−1^)	5.57 × 10^4^			1.64 × 10^5^			2.50 × 10^5^			4.05 × 10^5^		
*K_q_* (M^−1^ s^−1^)	5.57 × 10^12^			1.64 × 10^13^			2.50 × 10^13^			4.05 × 10^13^		
*K_α_* (M^−1^)	2.63 × 10^12^			3.97 × 10^14^			6.41 × 10^15^			1.43 × 10^15^		
*n*	2.99			3.05			3.26			3.04		

## Data Availability

The original contributions presented in the study are included in the article, further inquiries can be directed to the corresponding author.

## Abbreviations

ASE, accelerated solvent extraction; HPAEC, high-performance anion-exchange chromatography; HPGPC, high-performance gel permeation chromatography; DLS, dynamic light scattering; FT-IR, Fourier transform-infrared spectroscopy; TGA, thermal gravimetric analysis; XRD, X-ray diffraction; SEM, scanning electron microscopy; HG, homogalacturonan; RG-I, rhamnogalacturonan-I; Mw, weight-average molecular weight; Mn, number-average molecular weight.
